# A survey of extracorporeal photopheresis treatment in pediatric patients in the United Kingdom

**DOI:** 10.1002/jha2.58

**Published:** 2020-07-13

**Authors:** Aisling M. Flinn, Sheba Macheka, Mary Slatter, Anna‐Maria Ewins, Brenda Gibson, Sarah Lawson, Anna Tailby, Giovanna Lucchini, Helen New, Beki James, Arun Alfred, Julia Scarisbrick, Andrew R Gennery

**Affiliations:** ^1^ Department of Paediatric Immunology and Haematopoietic Stem Cell Transplantation Great North Children's Hospital Newcastle upon Tyne UK; ^2^ Haematology/Oncology Department The Royal Hospital for Children Glasgow UK; ^3^ Department of Haematology Birmingham Children's Hospital Birmingham UK; ^4^ BMT Department Great Ormond Street Hospital London UK; ^5^ NHS Blood and Transplant London Imperial College London UK; ^6^ Regional Centre for Paediatric Haematology Leeds Children's Hospital Leeds UK; ^7^ Department of Photopheresis Rotherham Foundation Trust Rotherham UK; ^8^ Department of Dermatology University Hospital Birmingham Birmingham UK

**Keywords:** extracorporeal photopheresis, GVHD, immunosuppression, pediatrics, transplantation

## Abstract

Extracorporeal photopheresis (ECP) is a second‐line therapy in acute and chronic GVHD and solid organ transplant rejection. We report ECP use in 98 pediatric patients in seven UK centers from 2010 to 2017, the majority treated for aGVHD (73.5%). ECP was safe and well tolerated including in low body weight patients. Most patients were on multiple immunosuppressive therapies prior to ECP; 45.9% were able to reduce or stop immunosuppression with treatment. Complete or partial response was reported in almost 60%. This study supports the need to include ECP treatment data to national transplant databases to provide accurate information regarding service provision, patient outcomes, and safety.

## 1 INTRODUCTION

Extracorporeal photopheresis (ECP) involves the collection of peripheral blood mononuclear cells by apheresis, exposure to photoactive 8‐methoxypsoralen and ultraviolet A radiation, and re‐infusion of the photoactivated cells into the patient [1]. Initially advocated for cutaneous T‐cell lymphoma treatment [2], it has since shown to be effective in acute and chronic graft‐versus‐host disease (aGVHD, cGVHD) and solid organ rejection [3, 4]. Results of ECP treatment in aGVHD are encouraging with a response rate of almost 70% in all affected organs reported [5]. It is recommended as a second‐line therapy in aGVHD and cGVHD [4, 5].

The major advantage of ECP is its lack of global immunosuppression, thereby not increasing the risk of serious infections or disease relapse or interfering with the graft‐versus‐leukemia effect [6, 7]. In addition, several studies have demonstrated the corticosteroid‐sparing benefits [8, 9]. Adverse side effects of ECP are minimal, predominantly related to central venous access. Children can develop hypovolemia, although this is less problematic with red blood cell priming of the circuit and development of the Therakos Cellex^®^ continuous flow system. Barriers to ECP treatment include access as it is available in a limited number of hospitals, cost, and the need for long‐term central venous access.

Pediatric ECP in the United Kingdom was established in 2010 in Rotherham General Hospital with the Therakos Cellex^®^ system permitting treatment in patients <40 kg. There are currently 11 UK pediatric hematopoietic stem cell transplant (HSCT) centers with variable access to ECP. Our aim was to document the extent of pediatric ECP use, particularly following NHS England approval of ECP as second‐line treatment for aGVHD and cGVHD in 2017, and provide an overview of treatment indications, outcomes, and safety.

## 2 METHODS

A survey developed by UK Photopheresis Society members was sent to 10 UK centers known to carry out pediatric ECP. For each patient, the following was requested: gender, underlying diagnosis, type of transplant, age at transplant, indication for ECP (corticosteroid refractory disease, corticosteroid dependence or intolerance), age/weight at ECP commencement, concurring immunosuppression, number of ECP cycles, type of venous access, whether blood priming of the ECP circuit was used, anti‐coagulation used, side effects of ECP, change in immunosuppression, and outcome (no [NR], partial [PR], or complete response [CR] or ongoing). For patients with aGVHD, the following were collected: organ involvement and overall disease grade.

## 3 RESULTS

A total of 98 pediatric patients (69 males, 29 females) were identified who received ECP between 2010 and 2017 in seven UK centers. The majority received treatment at Rotherham General Hospital (n = 45), followed by the Great North Children's Hospital Newcastle upon Tyne (n = 25), Birmingham Hospital (n = 11), Leeds Children's Hospital (n = 6), Great Ormond Street Hospital (n = 6), Glasgow Hospital (n = 4), and St Mary's Hospital, London (n = 1).

Ninety‐one of 98 (92.9%) patients had undergone allogeneic HSCT (allo‐HSCT). The indication for allo‐HSCT was hematological malignancy in 46 of 91 (50.6%) cases; primary immune deficiency in 21 of 91 (23.1%) cases; a non‐malignant hematological condition in 19 of 91 (20.9%) cases, and four of 91 (4.4%) cases had a metabolic condition. Underlying diagnosis was unknown in one case. Seven of 98 (7.1%) patients had undergone solid organ transplant; one lung, two liver, two liver and small bowel, and two small bowel transplants. Mean age at transplantation was 6.7 years (range 0.2‐18.4 years). Among those who underwent allo‐HSCT, 46 of 91 (50.6%) cases had a matched unrelated donor (5 bone marrow [BM], 5 peripheral blood [PB], 11 cord blood [CB], 25 not specified), 30 of 91 (32.9%) cases had a matched related donor (7 BM, 3 PB, 20 not specified), and six of 91 (6.6%) cases had a haploidentical donor (1 PB, 5 not specified). Three of 91 (3.3%) cases received CB but the donor type was not specified. Donor graft type was unknown for six (6.6%) cases.

Among patients who had undergone allo‐HSCT, 72 of 91 (79.1%) had aGVHD, 15 of 91 (16.5%) had cGVHD, and four of 91 (4.4%) had GVHD unspecified. All seven patients who had undergone solid organ transplant had rejection. Among those with aGVHD, the largest subgroup (27/72, 37.5%) had skin involvement only, 22 of 72 (30.6%) had two organ involvement, and 11 of 72 (15.2%) had three organ involvement (Table 1). Thirty‐nine of 72 (54.2%) had aGVHD maximum grade documented; 16 patients had grade 2, 14 had grade 3, and nine had grade 4. Prior to ECP, 87 of 98 (88.8%) received corticosteroids. Regarding immunosuppressive agents used before ECP (excluding continued prophylaxis), six received none, 21 received one, 43 received two, 22 received three, and six patients received four to six. Immunosuppression used included corticosteroids, ciclosporin, mycophenolate mofetil, tacrolimus, infliximab, basiliximab, imatinib, sirolimus, ruxolitinib, alemtuzumab, and anti‐thymocyte globulin. Three patients received mesenchymal stem cells. Forty‐four of 98 (44.9%) also continued on immunosuppressive prophylaxis, most commonly ciclosporin and/or mycophenolate mofetil.

**TABLE 1:** Organ involvement in patients with acute GVHD

Acute GVHD organ involvement	Number of patients	Response to ECP
Skin alone	27	CR 13 (48.2%), PR 4 (14.8%), NK 8 (29.6%), NR 2 (7.4%)
Skin and GIT	17	CR 7 (41.2%), PR 6 (35.3%), NK 4 (23.5%)
Skin and liver	1	NK 1 (100%)
GIT alone	7	CR 4 (57.1%), NK 3 (42.9%)
GIT and liver	1	CR 1 (100%)
Liver alone	3	CR 2 (66.7%), NK 1 (33.3%)
Lung alone	1	NK 1 (100%)
Lung and skin	2	PR 1 (50%), NK 1 (50%)
Lung and GIT	1	CR 1 (100%)
Skin, GIT and liver	11	CR 4 (36.4%), PR 1 (9.1%), NK 6 (54.5%)
Unknown	1	PR 1 (100%)

Abbreviations: GIT; gastrointestinal tract, CR; complete response, PR; partial response, NK; not known, NR; no response. Total number of patients with acute GVHD was 72.

ECP was indicated because of corticosteroid refractory disease in 25 (25.5%), corticosteroid dependence in 20 (20.4%), corticosteroid intolerance in two (2%), concurrent infections in two (2%), and was not documented for 49 (50%) patients. Mean age at starting ECP was 7.9 years (range 0.34‐18.6 years), three were <1 year. Mean weight was 27.5 kg (range 6.8‐93 kg), 12 weighed ≤10 kg. All patients received ECP via central venous access. Eighty‐five of 98 (86.7%) required blood priming of the ECP circuit, six (6.1%) did not (unknown for 7). Anticoagulation was with heparin in 66 (67.3%) and anticoagulant citrate dextrose solution in 25 (25.5%) (unknown for 7). Mean number of ECP cycles among all patients was 17 (range 1–89 cycles). Among survivors who had a partial or complete response, and completed treatment, the mean number of ECP cycles was 22 (range 6–89). Regarding side effects, five of 98 (5.1%) had complications associated with central venous access (4 with line infections, details not provided for 1). One patient had thrombocytopenia, although the suspected cause was not provided. No other ECP‐related adverse effects were reported.

At the time of data collection, treatment was ongoing for 18 (18.4%), 30 (30.6%) had died, and 50 (51%) had stopped ECP and were alive. CR was reported in 42 of 98 (42.9%), PR in 16 of 98 (16.3%), and NR in 6 of 98 (6.1%). Response was not reported in 13 (13.3%) for whom treatment was ongoing, 19 (19.4%) who had died, and in two (2%) who had stopped treatment but were alive. Among patients with aGVHD, 32 of 72 (44.4%) had a CR and 13 of 72 (18.1%) had a PR (NR in 2/72, not known in 25/72). In skin only aGVHD, CR or PR was reported in 63% (Table 1). In cGVHD, six of 15 (40%) had a CR and two of 15 (13.3%) had a PR (NR in 3/15, not known in 4/15). In patients with solid organ rejection, only two of seven were alive and had responded to therapy. Five of seven patients had died, although two of these patients were reported to have responded to ECP.

Ten of 98 (10.2%) ceased concomitant immunosuppressive therapy while on ECP, 35 of 98 (35.7%) reduced immunosuppression, and it remained unchanged for 25 of 98 (25.5%). Three (3.1%) had to increase immunosuppression. Change in immunosuppression was unknown for 22 of 98 (22.5%) and deemed too early to judge as treatment was ongoing for three (3.1%). Among the 30 patients who died, the reason for death was known for nine (5 from infection, 1 from GVHD, 2 from disease relapse, and 1 from pulmonary fibrosis). Among patients ≤10 kg, six of 12 (50%) died (2 from sepsis, 1 from disease relapse, 3 unknown), and four of these patients were on ≥3 immunosuppressive agents. Of those ≤10 kg who were alive, three had a CR, one PR, one NR, and treatment was ongoing for one.

## 4 DISCUSSION

This survey provides important insight into delivery of pediatric ECP in the United Kingdom. Most patients received ECP with minimal side effects, reinforcing the evidence that it is safe and well tolerated in children, including those with a low body weight. The majority were on multiple immunosuppression prior to ECP, highlighting the vulnerability of this population. This reflects that traditionally ECP has been used later in disease management, mainly due to issues with treatment access. ECP is increasingly available and used earlier in the disease course, important as early ECP initiation is associated with better clinical outcomes [8]. The number and wide range of different immunosuppressive therapies used highlight the varying approach and lack of evidence available in the management of corticosteroid refractory/dependent disease, underscoring the importance of continued research to determine the optimal second line treatment strategy. These data also demonstrate the corticosteroid‐sparing effects of ECP, with 45 of 98 (45.9%) stopping or reducing their immunosuppression.

There were limitations. Due to the snapshot nature of the survey, it included patients on ongoing treatment therefore not capturing the full impact of completed ECP on all patients. Important aspects of data were incomplete including ECP indication (unknown in 50%) and cause of death (unknown in 70%). 30 patients in this cohort died, including 50% of patients ≤10 kg, the majority who were on significant immune suppression, again highlighting the vulnerability of this population and the need to find ways to improve their management and clinical outcomes. Identifying gaps in our knowledge regarding management of this patient cohort supports the need to include ECP treatment data to national transplant databases. There is a lack of data to inform clinicians regarding patient selection, treatment schedules, and monitoring protocols for ECP. Cohesive and complete prospective patient data collection is essential to provide accurate information regarding service provision, patient outcomes, and safety as well as being a valuable tool in conducting research.

## CONFLICT OF INTEREST

The authors have no conflicts of interest to declare.

## Data Availability

The data that support the findings of this study are available from the corresponding author upon reasonable request.
